# Multi-Modal Joint Pulsed Eddy Current Sensor Signal Denoising Method Integrating Inductive Disturbance Mechanism

**DOI:** 10.3390/s25123830

**Published:** 2025-06-19

**Authors:** Yun Zuo, Gebiao Hu, Fan Gan, Zhiwu Zeng, Zhichi Lin, Xinxun Wang, Ruiqing Xu, Liang Wen, Shubing Hu, Haihong Le, Runze Wu, Jingang Wang

**Affiliations:** 1 Construction Branch State Grid Jiangxi Electric Power Co., Ltd., Nanchang 330036, China; yz4547339@sina.com (Y.Z.); hugebiao@proton.me (G.H.); fg9514553@proton.me (F.G.); zwzeng21@163.com (Z.Z.); zl84315113@sina.com (Z.L.); xw7383589@sina.com (X.W.); xuruiqing103@126.com (R.X.); 2 Yichun Power Supply Branch of State Grid Jiangxi Electric Power Co., Ltd., Yichun 336000, China; wenliang_0901@proton.me; 3 China Power Construction Group Jiangxi Electric Power Design Institute Co., Ltd., Nanchang 330046, China; hushubing@proton.me (S.H.); jxepdi-lehaihong@powerchina.cn (H.L.); 4 State Key Laboratory of Power Transmission Equipment Technology, School of Electrical Engineering, Chongqing University, Chongqing 400044, China; 15517775920@163.com

**Keywords:** pulsed eddy current signal, improved whale optimization, inductive disturbance mechanism, sensor signal processing, IWOA-VMD-SVD-WTD

## Abstract

Pulsed eddy current (PEC) testing technology has been widely used in the field of non-destructive testing of metal grounding structures due to its wide-band excitation and response characteristics. However, multi-source noise in industrial environments can significantly degrade the performance of PEC sensors, thereby limiting their detection accuracy. This study proposes a multi-modal joint pulsed eddy current signal sensor denoising method that integrates the inductive disturbance mechanism. This method constructs the Improved Whale Optimization -Variational Mode Decomposition-Singular Value Decomposition-Wavelet Threshold Denoising (IWOA-VMD-SVD-WTD) fourth-order processing architecture: IWOA adaptively optimizes the VMD essential variables (K, α) and employs the optimized VMD to decompose the perception coefficient (IMF) of the PEC signal. It utilizes the correlation coefficient criterion to filter and identify the primary noise components within the signal, and the SVD-WTD joint denoising model is established to reconstruct each component to remove the noise signal received by the PEC sensor. To ascertain the efficacy of this approach, we compared the IWOA-VMD-SVD-WTD method with other denoising methods under three different noise levels through experiments. The test results show that compared with other VMD-based denoising techniques, the average signal-to-noise ratio (SNR) of the PEC signal received by the receiving coil for 200 noise signals in different noise environments is 24.31 dB, 29.72 dB and 29.64 dB, respectively. The average SNR of the other two denoising techniques in different noise environments is 15.48 dB, 18.87 dB, 18.46 dB and 19.32 dB, 27.13 dB, 26.78 dB, respectively, which is significantly better than other denoising methods. In addition, in practical applications, this method is better than other technologies in denoising PEC signals and successfully achieves noise reduction and signal feature extraction. This study provides a new technical solution for extracting pure and impurity-free PEC signals in complex electromagnetic environments.

## 1. Introduction

Pulsed eddy current (PEC) testing technology is an electromagnetic non-destructive testing technology developed in recent decades. It is used for defect detection and positioning of metal conductive components, conductivity measurement, stress detection, etc. [1,2]. It uses a pulse excitation signal. Compared with the harmonic eddy current detection technology using single-frequency or multi-frequency excitation, the detection signal has richer frequency components. At the same time, the output signal of the pulsed eddy current sensor is mainly expressed in the time domain. Compared with the single impedance expression of the harmonic eddy current, the feature quantity that can be extracted is greatly expanded [3]. However, since the mid- and late-stage PEC signals are easily interfered with by noise in the form of industrial frequency, random, and artificial noise, this noise will make the measured PEC signal not pure enough, seriously affecting the strength and accuracy of signal detection.

To successfully mitigate noise interference and enhance detection accuracy, numerous scholars have developed various sensor signal denoising techniques [4]. The empirical mode decomposition (EMD) methodology introduced by Huang N E et al. [4] offers an innovative approach to sensor signal processing by disaggregating noisy PEC signals into intrinsic mode functions (IMFs) over several temporal scales. Nevertheless, in real applications, the EMD approach encounters issues such as reciprocal interference across many modes, which compromises detection accuracy. To address these deficiencies, researchers have sequentially introduced enhanced algorithms, including ensemble empirical mode decomposition (EEMD) [5], complementary ensemble empirical mode decomposition (CEEMD) [6], and complete ensemble empirical mode decomposition with adaptive noise (CEEMDAN) [7], which offer more efficacious solutions for PEC sensor signal denoising. Despite the enhancements in the aforementioned methods, the efficacy of noise reduction remains insufficient to entirely eradicate noise interference. To resolve this technical impediment, Dragomiretskiy and Zosso [8] introduced the variational mode decomposition (VMD) technique. This technique integrates the noisy signal with the variational framework, addressing the intrinsic limitations of the EMD method by identifying the optimal solution within the variational model. The efficacy of VMD is significantly influenced by parameter configurations, particularly the choice of the decomposition layer number K and the penalty factor α. To enhance these critical parameters, certain researchers employ swarm intelligence optimization methods for parameter refinement, hence enhancing the noise reduction efficacy of VMD. For example, Duan Xueying et al. [9] used VMD with improved particle swarm optimization (PSO) to predict power load; Liu Diyang et al. [10] used the dung beetle optimization (DBO) algorithm to optimize VMD parameters and combined it with neural networks to diagnose bearing faults; Zhang et al. [11] employed the Harris hawk optimization (HHO) approach for optimum variational mode decomposition (VMD) parameters and denoise transient electromagnetic power frequency data, yielding favorable outcomes. With the emergence of a large number of algorithms, Mirjalili and Seyedali developed a new optimization algorithm, namely WOA [12], which is a meta-heuristic optimization technique derived from the repeated search principles exhibited by humpback whale groups. It utilizes the bubble net hunting approach of humpback whales to effectively solve difficult optimization problems by mimicking their predatory behavior. Nonetheless, conventional algorithms exhibit significant constraints in population initialization methodologies and global optimization efficacy. In response to these limitations, Zhao et al. [13] used circular chaos mapping to enhance the sparrow search algorithm (SSA) to avoid the algorithm falling into local optimality. Nguyen et al. [14] integrated the random walk method with the grey wolf optimization (GWO) algorithm, significantly enhancing the program’s global search efficacy and preventing the search process from becoming trapped in local optima.

When processing PEC signals affected by multi-source noise, the single VMD method shows some limitations. The quality of the denoising effect often depends on whether the denoising method selected for different types of noise is appropriate. To solve this problem, singular value decomposition (SVD) was introduced, which is a technology that can process full-band noise [15]. Jing-Yi L et al. [16] suggested a wavelet threshold denoising (WTD) approach to mitigate Gaussian white noise in the signal by differentiating the amplitudes of both the signal and the noise within the wavelet domain. In 2021, Qi Tingye et al. [17] proposed a sensor signal denoising method based on WOA-VMD for processing transient electromagnetic signals. The following year, Qi Tingye et al. [18] proposed a VMD-WTD pulse eddy current sensor signal denoising method that combines VMD and WTD based on the previous method. In addition, Lv P et al. [19] effectively attenuated multi-source noise in the semi-airborne transient electromagnetic method by fusing WTD and adaptive singular value decomposition filtering. However, whether it is a single SVD or a single WTD, its denoising effect depends largely on the selection of singular value parameters or wavelet thresholds, which often leads to suboptimal results. In 2024, Song et al. [20] integrated VMD, SVD, and WTD for the first time, introducing a VMD fusion technique based on enhanced DBO, referred to as the VMD-SVD-WTD fusion algorithm. However, this method lacks a certain local search capability in model optimization and may fall into a local optimal solution.

This study offers a multimodal joint denoising approach, VMD-SVD-WTD, utilizing the improved WOA algorithm to address the aforementioned issues. This approach presents the inductive disturbance mechanism [21] and substitutes the conventional logarithmic spiral structure with an equidistant Archimedean spiral. The IWOA is obtained by improving the whale algorithm and optimizing the number of modes K and the penalty parameter α in the VMD algorithm. Subsequently, VMD decomposition is executed on the pulsed eddy current signal utilizing the derived optimum parameters to produce K IMF components. Following that, these IMF components are structured into a Hankel matrix, followed by the execution of SVD decomposition to eliminate the full-band noise. The remaining noise is ultimately treated with WTD to reassemble the denoised signal. This technique improves the local search efficacy of the conventional WOA method and prevents the model from converging to a preferred location during the optimization phase, consequently influencing the effectiveness of the VMD parameters. To improve the detection accuracy of pulse eddy current signals, a new solution is provided.

## 2. Using Improved Whale Optimization to Construct Variational Mode Decomposition Parameter Selection

### 2.1. Improved Whale Optimization (IWOA)

WOA is inspired by the unique hunting strategy of humpback whales and has powerful global search and optimization capabilities. However, its local search mechanism has certain limitations and is often prone to falling into local optimal states. To solve this problem, an inductive disturbance mechanism is added to WOA to improve its local search performance. In addition, we also replace the classic logarithmic spiral curve with a constant-pitch Archimedean spiral curve, which effectively reduces the risk of local optimization.

### 2.2. Whale Optimization Improvement Method

#### 2.2.1. Inductive Disturbance Mechanism

To improve the nearby search efficacy of the WOA algorithm, a collection of more advantageous search places X*(*t*) is produced following each iteration. Instead of immediately advancing to the next iteration, the algorithm first applies a perturbation factor to comprehensively investigate the vicinity of X*(*t*) and thereafter produces a new optimum search agent in later iterations. This is the best candidate solution found by the algorithm so far during the iteration process, representing the position of the current optimal solution. To address the deficiencies in randomness and directionality of the perturbation approach, the perturbation range is predetermined during the construction of the perturbation factor to guarantee the precision of the local search. In addition, a perception coefficient should be introduced to dynamically adjust the search agent’s concentration within the disturbance range, so that it can more accurately lock the potential optimal area near the current optimal solution [21]. The search agent consistently modifies the perturbation range and substitutes the prior value with the current optimum value to obtain the desired outcome. The numerical representation of the perturbation factor is as follows:

(1) $$\varepsilon =\frac{x_{d}-x}{\left||x_{d}-x|\right|}\cdot Step\cdot Rand(\cdot )$$

(2) x=x+u⋅Rand(⋅)⊕ε

Formula (2) denotes the ultimate position of the agent search among disturbance interference. In the formula, *u* denotes the coefficient that specifies the disturbance distance, ***Rand***(∙) represents a random number within the interval (−1, 1), *Step* indicates the movement increment of the search agent during the disturbance, *x_d_* signifies the position at time *d*, *x* refers to the optimal position, ⊕ denotes pointwise multiplication, and *x_d_* − *x*/|| *x_d_* − *x*|| illustrates the characteristic of the fitness function.

#### 2.2.2. Search Path Changes

The Archimedean spiral, or constant speed spiral, is a path created by a point that moves away from a fixed point at a uniform speed while simultaneously rotating around it at a constant angular velocity. The original method has a progressive variation in the pitch of the spiral, which influences its performance. To enhance performance, we select an Archimedean spiral with a constant pitch to maintain a fixed pitch, and we incorporate a correction coefficient to modify the spiral’s radius. The introduction of the correction coefficient enhances the function parameters by dynamically adjusting the spiral’s radius and its periodic fluctuations, thereby optimizing the algorithm’s balance between global exploration and local exploitation for superior convergence performance. The mathematical representation of the search trajectory is illustrated in Formula (3).

(3) $$\left\{\begin{array}{c} x=[(a+b\cdot l)\cdot (1+\delta \cdot \sin(k\cdot \theta ))]\cdot \cos(2\pi l)\\ y=[(a+b\cdot l)\cdot (1+\delta \cdot \sin(k\cdot \theta ))]\cdot \sin(2\pi l) \end{array}\right.$$

Formula (3) is typically articulated in polar coordinates, where *l* represents a random variable within the interval [−1, 1], *θ* denotes the current offset angle, *a* signifies the initial position offset coefficient, *b* is an integer that constrains the circle’s dimensions, *δ* remains a parameter that regulates the correction intensity, and *k* is the coefficient for the correction period.

WOA is enhanced by the alteration of the search trajectory and the incorporation of a perturbation mechanism. The method must identify a superior location promptly during the initial phase of perturbation. During the last phase of perturbation, the search agent will perform a thorough examination of positions in proximity to the target. Furthermore, the step size of the search agent is redefined as follows:

(4) $$Step={Step}_{min}+({Step}_{max}-{Step}_{min})\frac{N-n}{N}$$

According to Formula (4), the *step* size progressively diminishes, attaining its largest value at the outset of the iteration and its minimum by the conclusion of the iteration. To ascertain the optimality of the WOA search trajectory, we juxtaposed it with three classical curves: the logarithmic curve, the rose curve, and the Fermat curve, and executed tests utilizing various test functions to validate the efficacy of the Archimedean spiral curve for parameter optimization. The test functions are shown in Equations (5)–(7):

(5) $$f(x)=\sum_{i=1}^{d}x_{i}^{2}$$

(6) $$f(x)=\sum_{i=1}^{d}\left|x_{i}\right|+\prod_{i=1}^{n}\left|x_{i}\right|$$

(7) $$f(x)=\sum_{i=1}^{d-1}(100(x_{i+1}-x_{i}{)}^{2}+(x_{i}-1{)}^{2})$$

**Figure 1:**
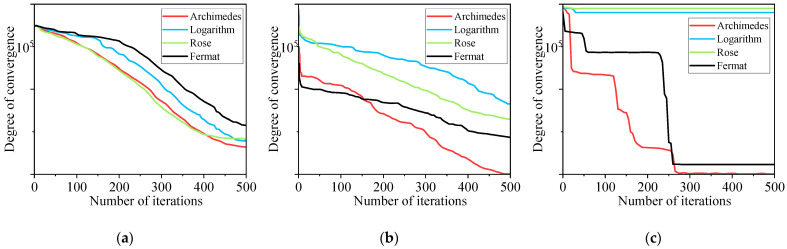
Search path comparison chart: (**a**–**c**) are the results of three different tests.

Figure 1 illustrates that, with an increase in iterations, all four curves converge. However, the rate at which convergence occurs for the WOA utilizing the Archimedean spiral as the search trajectory is markedly superior to that of the other three curve types, that is, the fastest convergence speed is obtained with fewer iterations. Numerous route search experiments have demonstrated the superiority of the Archimedean spiral curve for parameter optimization.

### 2.3. Improved Whale Optimization Implementation Steps

#### 2.3.1. Prey Hunting

Initially, the WOA algorithm regards the target prey as the optimal candidate, representing the most advantageous agent. Upon identifying the optimal agent, the remaining agents will realign their positions accordingly. The mathematical representation of this procedure is as follows:

(8) $$D=|C\cdot X^{*}(t)-X(t)|$$

(9) $$X(t+1)=X^{*}(t)-A\cdot D$$

(10) A=2a⋅r−a

(11) C=2⋅r

***D*** represents the distance vector between the optimal search agent and the standard agent, *t* denotes the total number of variations, ***A*** and ***C*** are coefficient vectors, X*(*t*) signifies the known optimal vector, ***X***(*t*) indicates the locations of the other search agents, where ***r*** is a random vector within the interval [0, 1].

#### 2.3.2. Bubble Net Attack Phase

Two primary methods may be derived from the hunting behaviors of humpback whales: the circle-forming method and the spiral updating method. An Archimedean spiral is constructed to rectify circular discrepancies between the locations of spiral updates, as seen below:

(12) $$X(t+1)=\left[D^{’}\cdot (bl)\cdot (1+\delta \cdot \sin(k\cdot \theta ))\right]\cdot \cos(2\pi l)+X^{*}(t)$$

*D*’ = |X* − ***X***(*t*)| denotes the distance that exists between the whale and its target.

According to the habits of humpback whales, humpback whale groups will show a collective contraction trend when hunting, gradually forming a circular or spiral shape to surround the prey. To enable the model’s formation, it is presumed that there exists a 50% likelihood of selecting between the contraction and encircling mechanism and the spiral update position for updating the whales’ positions throughout the optimization process. The mathematical framework is outlined as follows:

(13) $$X(t+1)=\left\{\begin{array}{c} X^{*}(t)A\cdot D+\varepsilon ,p<0.5,\;\;\;p<0.5\\ {}[D^{’}\cdot (bl)\cdot (1+\delta \sin(k\cdot \theta ))]\cdot \cos(2\pi l)+X^{*}(t),p\geq 0.5 \end{array}\right.$$

where *p* is a random number in [0, 1].

#### 2.3.3. Searching for Prey

At this juncture, to expand the search range, the search agents are arbitrarily dispersed, their locations are substituted with those of the optimally selected candidate agents, and interference elements are introduced. The model for mathematical description is:

(14) $$D=|C\cdot X_{rand}-X(t)|$$

(15) $$X(t+1)=X_{rand}-A\cdot D+\varepsilon$$

***X****_rand_* is a randomly picked position vector (random whale) from the present population. In each cycle, the interference factor is applied to meticulously examine the vicinity surrounding the agent. In the subsequent cycle, a new optimal search agent will be created to revise the position. When |***A***| ≥ 1, a random agent is chosen as the subsequent reference. When |***A***| is less than 1, the optimal search agent is chosen.

When using IWOA to enhance the choice of parameters for VMD, it is crucial to determine a suitable fitness value to evaluate the procedure. This study utilizes the least mean envelope entropy as the optimization objective function and subsequently evaluates the efficacy of IWOA against other optimization methods in improving VMD parameters. Consequently, the VMD decomposition optimization model predicated on the minimal mean envelope entropy may be articulated as:

(16) $$\&\underset{K,a}{\min}J(K,\alpha )=-\frac{1}{K}\sum_{i=1}^{K}\sum_{j=1}^{m}p_{i,j}{log}_{2}p_{i,j}\left\{\begin{array}{c} \begin{array}{c} s.t.K_{min}\leq K\leq K_{max}\\ \alpha_{min}\leq \alpha \leq \alpha_{max} \end{array} \end{array}\right.$$

In the aforementioned formula, *K* and α denote the quantity of decomposed modes and the quadratic penalty factor, respectively; *K_min_* and *K_max_* represent the lowest and highest values of the decomposed modes, respectively; *α_min_* and *α_max_* indicate the minimum and greatest values of the quadratic penalty factor, respectively; *p* signifies the signal probability distribution.

**Figure 2:**
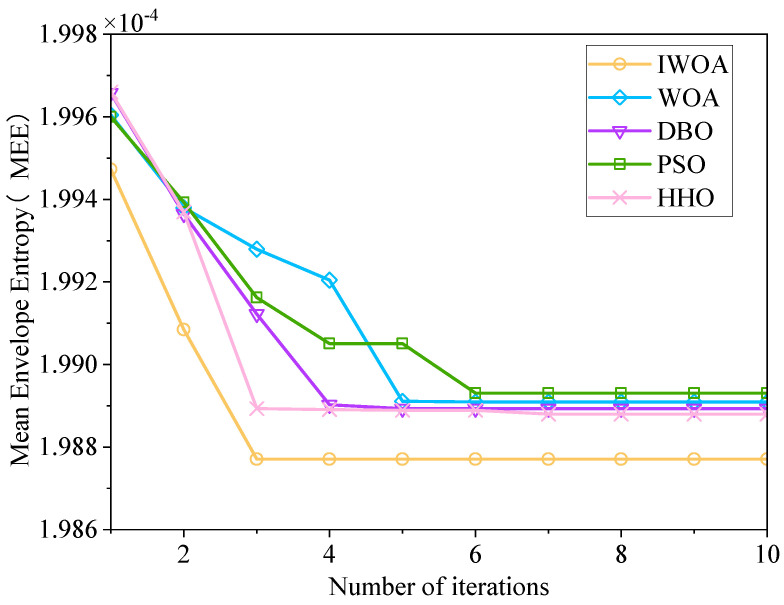
Convergence performance comparison chart.

The technique presented in this study outperforms previous optimization algorithms regarding optimization speed and noise levels. The convergence performance comparison chart is shown in Figure 2. It shows that the use of equal-pitch Archimedean path and the introduction of IWOA with perturbation mechanism can enhance the algorithm’s optimization ability and avoid falling into local optimality.

## 3. Variational Mode Decomposition-Singular Value Decomposition-Wavelet Threshold Denoising Combined Pulsed Eddy Current Noise Reduction Method Based on Improved Whale Optimization

### 3.1. Construction of Variational Mode Decomposition-Singular Value Decomposition-Wavelet Threshold Denoising Denoising Method

#### 3.1.1. VMD Decomposition Structure

The central frequency and bandwidth of each modal component are progressively refined to attain adaptive decomposition of the pulse eddy current signal frequency spectrum. This procedure may be considered a resolution method for a variational issue, whereby the restricted variational issue can be articulated as:

(17) $$\left\{\begin{array}{c} \underset{\left\{uk,\omega k\right\}}{min}\left\{\sum_{k=1}^{K}{\left\|\partial_{l}\left[\left(\delta (t)+\frac{j}{\pi t})*u_{k}(t\right)\right]e^{-j\omega_{k}t}\right\|}_{2}^{2}\right\}\\ \sum_{k=1}^{K}u_{k}(t)=f \end{array}\right.$$

where *u_k_* denotes the *K*th IMF component subsequent to VMD decomposition; *ω_k_* represents the instantaneous frequency of the *K*th IMF component; *δ*(*t*) signifies the Dirac function; (*δ*(*t*) + *j*/*πt*) ∗ *u_k_*(*t*) is the Hilbert transform.

To determine the best solution of the variational issue represented by Formula (17), the quadratic penalty factor *α* and the Lagrangian multiplier *λ* are introduced, leading to the construction of the augmented Lagrangian function:

(18) $$(\left\{u_{k}\right\},\left\{\omega_{k}\right\},\lambda )==a\sum_{k=1}^{K}{\left\|\partial_{l}\left[\left(\delta (t)+\frac{j}{\pi l})*u_{k}(t\right)\right]e^{-j\omega_{k}t}\right\|}_{2}^{2}\;+{\left\|f(t)-\sum_{k=1}^{k}u_{k}(t)\right\|}_{2}^{2}+\left\langle \lambda (t),f(t),\sum_{k=1}^{k}u_{k}(t)\right\rangle$$

#### 3.1.2. Singular Value Decomposition and Wavelet Threshold Denoising Construction

Singular value decomposition is used subsequent to VMD, utilizing the mean for every unique value as the threshold for further denoising of the full-band noise.

Each IMF component produced from VMD decomposition is restructured into a Hankel matrix format, followed by singular value decomposition to obtain its left singular vector matrix *U*, singular value matrix *σ*, and right singular vector matrix *V* [22]:

(19) $$H=U\Sigma V^{T}$$

where *H* is the Hankel matrix.

Following the SVD decomposition of the original Hankel matrix, the arithmetic mean *σ_i_* of the resultant singular value sequence is computed. A singular value screening procedure is conducted based on the threshold *σ_i_*: singular values equal to or over *σ_i_* are preserved, while those below *σ_i_* are nullified. The rebuilt Hankel matrix is ultimately acquired using this thresholding process:

(20) $$\Sigma^{’}=diag\left(\sigma_{1},\sigma_{2},\ldots ,\sigma_{i},0,0,\ldots ,0\right)$$

(21) $$H^{’}=U\Sigma^{’}V^{T}$$

**Figure 3:**
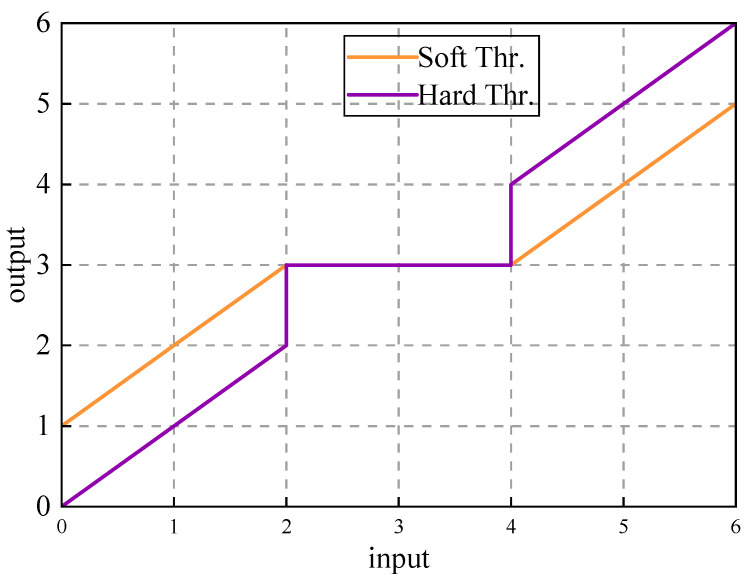
Soft and hard threshold function curve graph.

**Figure 4:**
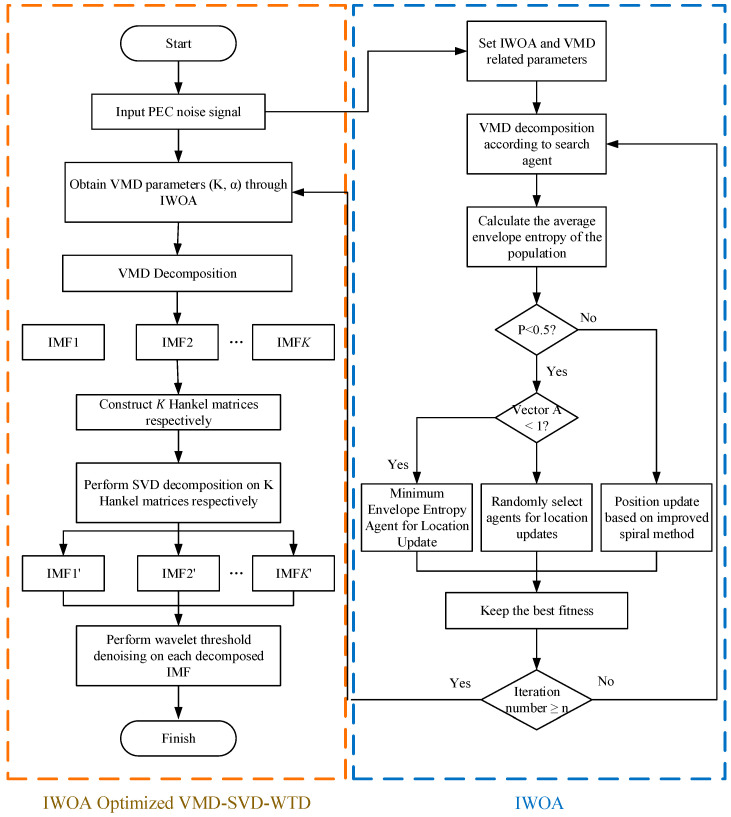
Diagram of the VMD-SVD-WTD pulsed eddy current noise elimination technique utilizing IWOA.

The sum of the negative diagonal elements of the reconstructed Hankel matrix is calculated, and the mean is taken as the characteristic parameter. The adaptive threshold is set by WTD technology, and the IMF component coefficients obtained by decomposition are divided into two categories: significant components and noise components: coefficients with absolute values exceeding the threshold are retained, while coefficients below the threshold are specially processed, thereby achieving accurate separation and reconstruction of noise signals [18]. Regarding the selection of the threshold function, considering the shortcomings of the hard threshold function that is easily affected by data fluctuations and lacks robustness, this study uses a soft threshold function with continuous characteristics to process the noise-dominated IMF components. The soft threshold function is as follows [23]:

(22) $$S_{\lambda }(x)=sign(x)\cdot max(|x|-\lambda ,0)$$

where *x* represents the data to be thresholded, *λ* represents the threshold parameter used to control the contraction intensity, and *sign*(*x*) represents the sign function. Figure 3 is a graph of a soft threshold function and a hard threshold function, and Figure 4 is a flow chart of a VMD-SVD-WTD pulsed eddy current denoising method based on IWOA.

### 3.2. Denoising Performance Indicators

This study uses signal-to-noise ratio (SNR), root mean square error (RMSE) and autocorrelation coefficient (AC) as the evaluation index system for denoising performance. These indicators reflect the denoising effect from different angles: SNR measures the degree of noise suppression, RMSE evaluates the accuracy of signal reconstruction, and AC represents the waveform similarity. The calculation formulas for each indicator are as follows:

(23) $$SNR=10\log\left(\begin{array}{c} \frac{\sum_{i=1}^{n}s_{i}^{2}}{\sum_{i=1}^{n}(s_{i}-y_{i}{)}^{2}} \end{array}\right)$$

(24) $$RMSE=\sqrt{\frac{1}{N}\sum_{i=1}^{n}(s_{i}-y_{i}{)}^{2}}$$

(25) $$AC=\frac{\sum_{i=1}^{n}\left(s_{i}-\overset{-}{s}\right)(y_{i}-\overset{-}{y})}{\sqrt{\sum_{i=1}^{n}(s_{i}-\overset{-}{s}{)}^{2}\cdot \sum_{i=1}^{n}(y_{i}-\overset{-}{y}{)}^{2}}}$$

In this evaluation framework, *s_i_* represents the original signal and *y_i_* denotes the denoised reconstructed signal. While SNR and RMSE typically show an inverse relationship (higher SNR generally corresponds to lower RMSE), it should be noted that these are independently calculated metrics reflecting different aspects of denoising performance. Similarly, a higher AC value indicates better waveform similarity between the reconstructed and original signals, demonstrating improved signal restoration quality.

## 4. IWOA-VMD-SVD-WTD Model Test

### 4.1. Device Structure and Parameter Settings

**Figure 5:**
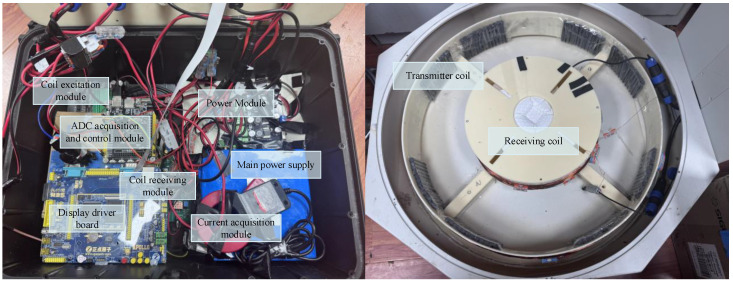
Schematic diagram of different modules of pulsed eddy current testing.

Before performing pulsed eddy current testing, a detailed investigation of the testing environment must be conducted to ensure the accuracy of parameter settings and the reliability of test results. The key steps include the following: (1) evaluating the material properties, geometry, surface state and potential interference factors of the object being tested; (2) setting coil parameters (size, shape, number of turns, lift-off distance) according to the test target; (3) selecting an appropriate sampling frequency to balance signal resolution and data processing efficiency; (4) optimizing input current parameters (amplitude, frequency, waveform) to achieve effective eddy current excitation. This prior information is crucial for selecting appropriate device parameters to obtain complete and accurate detection signals. Its integrity and accuracy lay a solid foundation for subsequent PEC noise reduction processing. Table 1 shows the device setting parameters, and Figure 5 is a schematic diagram of different modules of pulsed eddy current testing.

### 4.2. Random Noise Test

To assess the efficacy of our technique across varying signal-to-noise ratio situations, we established three distinct levels of random noise: high noise (SNR of −25 dB), moderate noise (SNR of −10 dB), and low noise (SNR of 5 dB). The IWOA-VMD-SVD-WTD model was employed to assess its denoising efficacy across three distinct noise levels.

**Figure 6:**
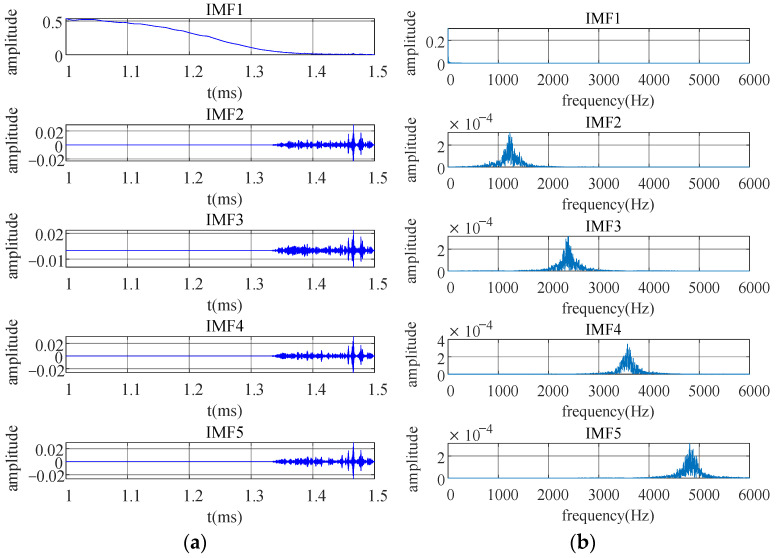
VMD waveform after decomposition. (**a**) Time domain diagram; (**b**) frequency domain diagram.

The best combination is achieved by improving the parameters, resulting in (K, α) = (5, 8000). Incorporating the PEC signal and these parameters into the denoising model will provide five modal components, as seen in Figure 6. Thereafter, the correlation coefficient for each component with the original signal is computed. The demarcation node is set with a threshold correlation value of 0.5. A correlation value of 0.5 signifies that the IMF component exhibits a robust association with the original signal. IMFs with a correlation coefficient below 0.5 demonstrate a diminished connection with the original signal. Table 2 reveals that the correlation coefficient of IMF1 is above the threshold of 0.5, signifying that this component is the primary signal component. The correlation coefficients of IMF2, IMF3, IMF4, and IMF5 fall below the 0.5 threshold, signifying that these components are predominantly influenced by noise and are primarily constituted of noise, whereas the IMF1 component is primarily governed by the PEC signal. A hybrid denoising technique for random noise is given based on the preceding study: the IMF1 component carrying valid signals is retained; for the IMF2–IMF5 components dominated by noise, a collaborative processing framework of SVD and WTD is constructed. SVD reconstructs the signal subspace by constructing the Hankel matrix and uses the multi-resolution analysis characteristics of WTD to adaptively process random noise at different scales.

**Figure 7:**
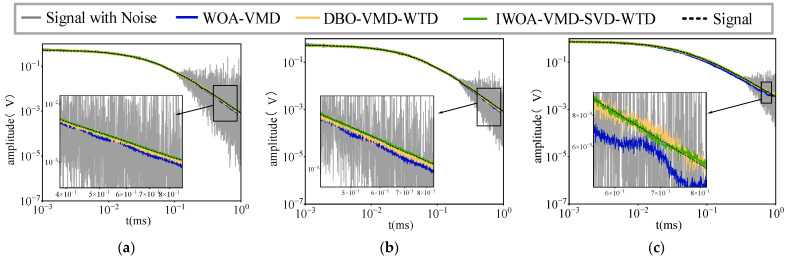
Suppression effect for different noise levels: (**a**) high noise (**b**) moderate noise (**c**) low noise.

Figure 7 presents the comparative findings of a series of noisy PEC signals prior to and after denoising. The figure clearly demonstrates that the signal waveform processed by the IWOA-VMD-SVD-WTD model exhibits considerable smoothness and greater consistency with the original signal, particularly in the presence of substantial noise interference. Upon evaluating the efficacy of various denoising techniques, the signal-to-noise ratio (SNR) subsequent to WOA-VMD, DBO-VMD-WTD, and IWOA-VMD-SVD-WTD processing is recorded at 15.48 dB, 26.36 dB, and 30.41 dB, respectively. This outcome conclusively demonstrates that the IWOA-VMD-SVD-WTD model not only efficiently mitigates noise but also preserves the essential components of the signal, therefore markedly enhancing its visibility and overall quality. This technique demonstrates enhanced robustness and adaptability in processing data in intricate noise settings.

Furthermore, we performed comparison studies utilizing the IWOA-VMD-SVD-WTD model alongside the WOA-VMD model and the DBO-VMD-WTD model, meticulously documenting the signal-to-noise ratio data post-denoising. The mean signal-to-noise ratio of 200 denoised signals is computed, as presented in Table 3. The experimental findings indicate that the IWOA-VMD-SVD-WTD model has considerable superiority in denoising efficacy.

**Figure 8:**
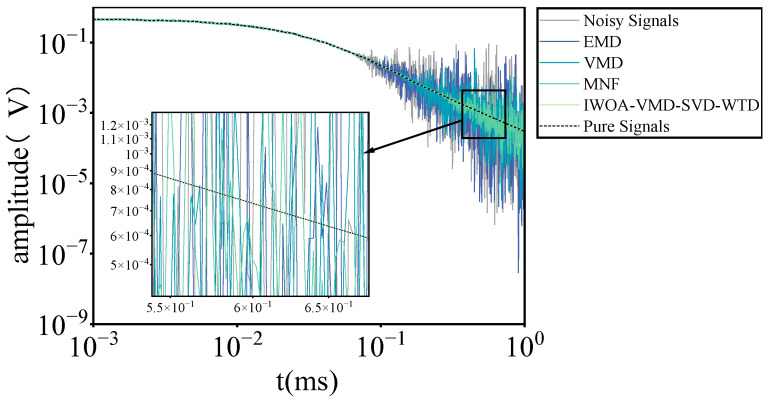
Comparison between conventional denoising methods and IWOA-VMD-SVD-WTD denoising.

To further ascertain the superiority of the traditional denoising method over the WOA-VMD model and to evaluate the comparative efficacy of the DBO-VMD-W method against the IWOA-VMD-SVD-WTD denoising technique, we conducted a series of comparative experiments utilizing several widely recognized denoising methodologies, including EMD, VMD, and minimum noise separation transform (MNF). Figure 8 presents the experimental data, distinctly illustrating the variances in denoising efficacy across the various approaches. The experimental results indicate that the IWOA-VMD-SVD-WTD technique greatly outperforms existing denoising methods, notably demonstrating exceptional denoising efficacy in the latter processing stages. The signal-to-noise ratios (SNRs) following processing with EMD, VMD, MNF, and IWOA-VMD-SVD-WTD are −7.91 dB, −7.43 dB, 2.86 dB, and 16.72 dB, respectively. The comparison of the signal-to-noise ratios indicates that the IWOA-VMD-SVD-WTD approach has markedly enhanced the denoising efficacy, hence underscoring its superiority in noise suppression.

### 4.3. On-Site Noise Test

This study utilized noise data acquired on-site at a substation to empirically validate the efficacy of the suggested approach in power non-destructive testing. By integrating the original PEC signal with the recorded noise from the substation, a noisy PEC signal sample of practical engineering relevance was created to emulate the signal acquisition conditions in an actual detection setting. To comprehensively assess the denoising efficacy of various approaches, the experiment chose three models: WOA-VMD, DBO-VMD-WTD, and IWOA-VMD-SVD-WTD, as presented in this study, for comparative analysis.

**Figure 9:**
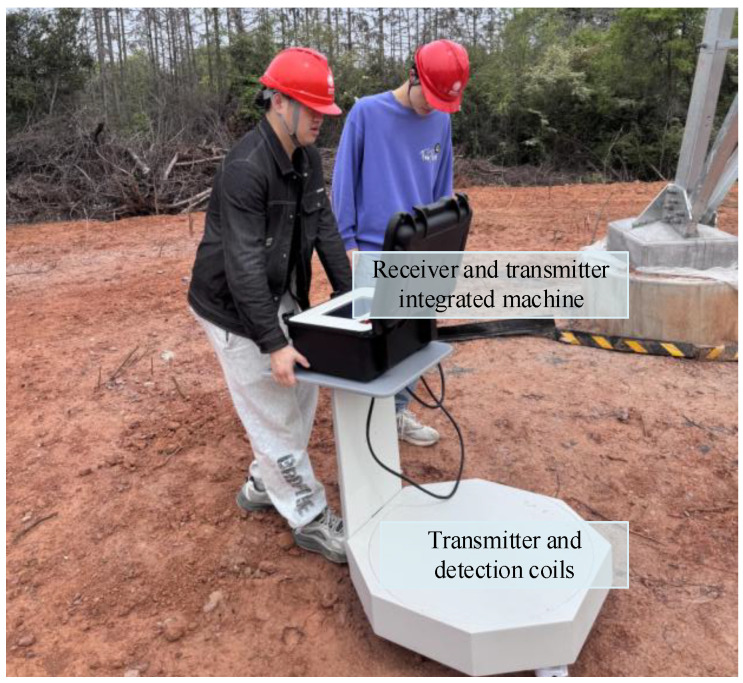
Actual photos of noise reduction detection by pulse eddy current device at substation site.

**Figure 10:**
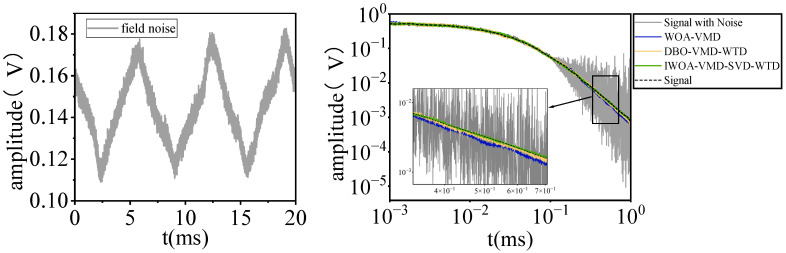
Noise suppression effects of three methods in the field.

Figure 9 is a real shot of the denoising detection of the pulse eddy current device at the substation. Figure 10 illustrates the random noise signal recorded on-site and the attenuation effect of the three models on the noise at the substation location. Table 4 thoroughly assesses the noise reduction efficacy using three quantitative metrics: SNR, RMSE, and AC.

The experimental results show that compared with the WOA-VMD and DBO-VMD-WTD models, the IWOA-VMD-SVD-WTD model exhibits better noise suppression ability, and its SNR value is significantly improved, reaching 31.7% and 19.2%, respectively; at the same time, the RMSE is reduced to 0.017, which is about 45.2% and 26.1% less than the other two methods; the AC index reaches 0.95, which is highly consistent with the original PEC signal. Waveform comparison analysis shows that the IWOA-VMD-SVD-WTD model can effectively filter out high-frequency interference while retaining the signal feature details and effectively retain the main components of the PEC signal.

## 5. Conclusions

Aiming at the problem of random noise interference in pulsed eddy current detection signals in grounding electrode assessment, this paper proposes an adaptive denoising method based on the improved whale optimization algorithm. The inductive disturbance mechanism is used to optimize the search path of the whale optimization algorithm, and the optimal mode number and penalty factor of variational mode decomposition are quickly determined. The noisy signal collected by the sensor is decomposed into multiple intrinsic mode components, and the signal-dominant component and the noise-dominant component are determined according to the correlation coefficient (whether it is greater than 0.5). A combined denoising model of singular value decomposition and wavelet thresholding is established, and signal reconstruction is achieved through hierarchical processing. Experimental verification leads to the following conclusions:

(1) The comparative experiment of introducing strong, medium and weak random noises into the receiving coil signal shows that the denoising performance of the IWOA-VMD-SVD-WTD model for sensor signals is better than that of the WOA-VMD and DBO-VMD-WTD methods, and its denoising curve has the highest similarity with the original pulsed eddy current signal. After processing 200 sets of noisy sensor signals, the average signal-to-noise ratios reach 24.31 dB, 29.72 dB and 29.64 dB, respectively, which is significantly improved compared with the comparison model.

(2) Compared with the traditional EMD, VMD and MNF methods, under the same sensor test conditions, the proposed model significantly improves the signal-to-noise ratio of the receiving coil signal. The signal-to-noise ratios of the four strategies are 16.72 dB, −7.91 dB, −7.43 dB and 2.86 dB, respectively, which reflects the technical advantages of the proposed method in sensor signal noise reduction.

(3) The substation field noise test demonstrates that the model maintains robustness under intricate operational conditions: the SNR is enhanced by 31.7% and 19.2% relative to the comparative method, the RMSE is diminished to 0.017 (a reduction of 45.2% and 26.1%), and the AC remains consistently above 0.95. The above experiments show that this method can extract pure pulsed eddy current signals from the sensor, confirming its reliability in engineering applications.

## Figures and Tables

**Table 1:** Pulsed eddy current device parameter setting.

Parameter Name	Parameter Value	Unit
Transmitter coil radius	0.2	m
Receiving coil radius	0.6	m
Transmitting coil turns	10	turn
Receiving coil turns	1700	turn
Sampling frequency	25	MHz
Excitation current amplitude	20	A
Excitation voltage	12	V
Excitation voltage waveform	Bipolar pulse square wave	/

**Table 2:** Correlation coefficients of different modal components.

Modal Components	IMF1	IMF2	IMF3	IMF4	IMF5
Correlation coefficient	0.8463	0.4648	0.3791	0.3119	0.1179

**Table 3:** Average signal-to-noise ratio of 200 denoised signals under three methods.

SNR	WOA-VMD	DBO-VMD-WTD	IWOA-VMD-SVD-WTD
−25	15.48	19.32	**24.31**
−10	18.87	27.13	**29.72**
5	18.46	26.78	**29.64**

**Table 4:** Field denoising indicators of three methods.

Indicator Function	WOA-VMD	DBO-VMD-WTD	IWOA-VMD-SVD-WTD
SNR (dB)	30.21	33.47	**39.78**
RMSE	0.031	0.023	**0.017**
AC	0.79	0.0.87	**0.95**

## Data Availability

The data used in the analysis presented in the paper will be made available, subject to the approval of the data owner.

## References

[1] Yang B., Luo F. (2004). Study of Pulsed Eddy Current Nondestructive Testing Technology. Instrum. Technol. Sens..

[2] Zhou D., Tian G., Wang H., Wang P. (2010). Evaluation of applied stress using pulsed eddy current technology. Chin. J. Sci. Instrum..

[3] Wu X., Zhang Q., Shen G. (2016). Review on advances in pulsed eddy current nondestructive testing technology. Chin. J. Sci. Instrum..

[4] Huang N.E., Shen Z., Long S.R., Wu M.C., Shih H.H., Zheng Q., Nai-Chyuan Y., Tung C.C., Liu H.H. (1998). The empirical mode decomposition and the Hilbert spectrum for nonlinear and non-stationary time series analysis. Proc. R. Soc. London. Ser. A Math. Phys. Eng. Sci..

[5] Wu Z., Huang N.E. (2009). Ensemble empirical mode decomposition: A noise-assisted data analysis method. Adv. Adapt. Data. Anal..

[6] Yeh J.R., Shieh J.S., Huang N.E. (2010). Complementary ensemble empirical mode decomposition: A novel noise enhanced data analysis method. Adv. Adapt. Data. Anal..

[7] Torres M.E., Colominas M.A., Schlotthauer G., Flandrin P. A complete ensemble empirical mode decomposition with adaptive noise. Proceedings of the 2011 IEEE International Conference on Acoustics, Speech and Signal Processing (ICASSP).

[8] Dragomiretskiy K., Zosso D. (2013). Variational mode decomposition. IEEE Trans. Signal Process..

[9] Duan X., Li X., Chen W. (2022). Improved particles swarm optimization algorithm-based VMD-GRU methodfor short-term load forecasting. New Technol. Electr. Eng. Energy.

[10] Liu D., Zhang Q., Zhu G. (2024). Bearing Fault Diagnosis Based on Optimized VMD Parameters and VGG Models. Mach. Tools Hydraul..

[11] Zhang Y., Wu Y., Li L., Liu Z. (2023). A Hybrid Energy Storage System Strategy for Smoothing Photovoltaic Power Fluctuation Based on Improved HHO-VMD. Int. J. Photoenergy.

[12] Mirjalili S., Lewis A. (2016). The whale optimization algorithm. Adv. Eng. Softw..

[13] Zhao X., Zhang J., Long Q. (2023). Indoor visible light positioning method using ISSA-ELM neural network based on circle chaotic mapping. Acta Opt. Sin..

[14] Nguyen H., Bui X.N., Drebenstedt C., Choi Y. (2024). Enhanced Prediction Model for Blast-Induced Air Over-Pressure in Open-Pit Mines Using Data Enrichment and Random Walk-Based Grey Wolf Optimization–Two-Layer ANN Model. Nat. Resour. Res..

[15] Qian Z., Cheng L., Li Y. (2011). Noise reduction method based on singular value decomposition. J. Vib. Meas. Diagn..

[16] Jing-Yi L., Hong L., Dong Y., Yan-Sheng Z. (2016). A new wavelet threshold function and denoising application. Math. Probl. Eng..

[17] Qi T.Y., Wei H.R., Feng G.R., Zhang X., Yu C., Zhao D., Sun D. (2021). Denoising method of transient electromagnetic detection signalbased on WOA-VMD algorithm. J. Cent. South Univ. Sci. Technol..

[18] Qi T., Wei X., Feng G., Zhang F., Zhao D., Guo J. (2022). A method for reducing transient electromagnetic noise: Combination of variational mode decomposition and wavelet denoising algorithm. Measurement.

[19] Lv P., Wu X., Zhao Y., Chang J. (2022). Noise removal for semi-airborne data using wavelet threshold and singular value decomposition. J. Appl. Geophys..

[20] Song D., Feng G., Qi T., Wang H., Pan D., Zhang L. (2024). A new combined transient electromagnetic noise reduction method of VMD-SVD-WTD based on improved dung beetle optimization algorithm with multi-strategy fusion. Meas. Sci. Technol..

[21] Sun W., Wang J., Wei X. (2018). An improved whale optimization algorithm based on different searching paths and perceptual disturbance. Symmetry.

[22] Lei Z., Wang F., Li C. (2023). A denoising method of partial discharge signal based on improved SVD-VMD. IEEE Trans. Dielectr. Electr. Insul..

[23] Donoho D.L., Johnstone I.M. (1994). Ideal spatial adaptation by wavelet shrinkage. Biometrika.

